# A Retrospective Study on Temporal Trends in Mortality Related to Atrial Fibrillation and Chronic Obstructive Pulmonary Disease

**DOI:** 10.7759/cureus.95373

**Published:** 2025-10-25

**Authors:** Chaitanya Singh, Singathala Gnana Sree, Anand Prakash, Asees G Singh, Mathew Anil Chempakasseril, Mahalakshmi Desikan, Abhishek Singh Chauhan

**Affiliations:** 1 Anatomy, Dr. D.Y. Patil Medical College and Hospital, Pune, IND; 2 Internal Medicine, Institute of Medical Sciences, Banaras, IND; 3 Internal Medicine, Sri Ramachandra Institute of Higher Education and Research, Sri Ramachandra Medical College (SRMC), Chennai, IND; 4 Medicine, Kasturba Medical College, Manipal, IND; 5 Pharmacology, Believer's Church Medical College and Hospital, Thiruvalla, IND; 6 Internal Medicine, St. Richards Hospital, Chichester, GBR; 7 Community Medicine, Amrita School of Medicine, Faridabad, IND

**Keywords:** age-adjusted mortality rate, atrial fibrillation, cdc mcd, chronic obstructive pulmonary disease, retrospective study

## Abstract

Introduction: Atrial fibrillation (AF) is a major cause of mortality, and its association with chronic obstructive pulmonary disease (COPD) remains unexplored. Individuals with these diseases have increased mortality and morbidity rates.

Aims: This study aimed to analyze mortality trends and demographic disparities in AF with COPD as a contributing cause using the CDC Wide-Ranging Online Data for Epidemiologic Research (CDC WONDER) Multiple Causes of Death (MCD) database from 1999 to 2020.

Methodology: A retrospective observational study was conducted using the CDC WONDER MCD database to assess mortality trends in individuals aged 25 years and older in the United States from 1999 to 2020. The study included deaths where AF (International Classification of Diseases, 10th Revision (ICD-10): I-48) was listed as the underlying cause and COPD (ICD-10: J40-J44) as a contributing cause. Data were analyzed by urbanization, gender, race, geographic region, and place of death. Age-adjusted mortality rates (AAMR) and annual percentage change (APC) were calculated using statistical analysis software JoinPoint, version 5.4.0, developed by the National Cancer Institute, Bethesda, MD.

Results: A total of 37,738 deaths were recorded. The AAMR for AF with COPD increased over time from 1999-2004 (APC: 6.82) to 2004-2015 (APC: 8.37), and then it plateaued from 2015 to 2022 (APC: -0.15). The highest mortality was observed in females, White patients, and metropolitan areas. Temporal trends showed that the mortality increased with time and became steady after 2015; there is a wide disparity in the mortality in races, and more in people living in metropolitan areas.

Conclusions: This study highlights significant mortality trends increasing in all AF with COPD, and from 2015, it is at a steady rate, with disparities by gender, race, and location. Findings underscore the need for targeted prevention strategies and improved healthcare access.

## Introduction

Atrial fibrillation (AF) is the most common sustained cardiac arrhythmia, affecting over 37.5 million people globally, and its prevalence is expected to increase substantially in the coming decades due to aging populations and rising comorbidities such as obesity and hypertension [1]. In the United States, AF was listed as the underlying cause in approximately 26,077 deaths in 2020, according to CDC Wide-Ranging Online Data for Epidemiologic Research (CDC WONDER) data. It is a leading contributor to stroke, heart failure, and all-cause mortality. However, significant disparities in AF-related mortality exist: older adults, women, and White individuals are disproportionately affected, while Black and Hispanic populations may be underdiagnosed despite higher risk profiles for contributing comorbidities [2, 3]. Moreover, geographic differences reveal that rural populations face worse outcomes due to healthcare access barriers and higher cardiovascular risk burdens [4].

Chronic obstructive pulmonary disease (COPD), a progressive inflammatory airway disorder, is another major contributor to global mortality, ranked as the third leading cause of death worldwide by the WHO. In the United States, over 150,000 deaths annually are attributed to COPD [5]. COPD commonly coexists with cardiovascular diseases, and its relationship with AF has drawn increasing attention due to shared risk factors such as smoking, systemic inflammation, and aging. Research has shown that COPD can increase the risk of new-onset AF by over 30%, with more severe lung dysfunction correlating with higher arrhythmia burden [6]. Demographic disparities in COPD mortality are pronounced: men have historically had higher rates, but women now show rising trends due to shifts in smoking patterns and biological vulnerability [7]. Black Americans and individuals living in rural or low-income areas bear a disproportionate burden, reflecting socioeconomic and environmental inequities [5].

The pathophysiological interplay between AF and COPD is multifaceted. COPD contributes to atrial remodeling through mechanisms like chronic hypoxia, hypercapnia, systemic inflammation, and pulmonary hypertension, all of which predispose patients to arrhythmogenesis [8, 9]. Hypoxia, for instance, activates HIF-1α and VEGF pathways, driving atrial fibrosis via TGF-β1 and MMP-9 signaling [10]. Simultaneously, AF may worsen COPD outcomes by impairing cardiac output and exacerbating respiratory symptoms. Despite this clinical intersection, there remains a paucity of large-scale population-based studies examining AF as an underlying cause with COPD as a contributing cause of death. Existing literature often fails to address granular disparities across sex, race, geographic location, and urbanization status. This study aims to fill this gap by analyzing nationwide mortality trends using the CDC Multiple Causes of Death (MCD) database (1999-2020) [8]. Through a comprehensive retrospective analysis, we aim to identify high-risk subgroups and inform targeted public health interventions.

The aim and objective of our study is to assess mortality trends in AF where COPD is a contributing cause of death and to analyze the differences by sex, race, and geolocation to identify potential disparities in mortality patterns.

## Materials and methods

A retrospective original research study was conducted using the CDC WONDER MCD database [8]. This publicly available dataset contains de-identified death certificate information for all recorded deaths in the United States. Data extraction was performed on April 7^th^, 2025. Since CDC WONDER comprises de-identified, publicly accessible data, this study was classified as non-human participant research, and institutional ethics approval was not required.

Mortality data were extracted for the years 1999-2020, including individuals aged 25 years and older, as AF-related deaths in younger populations are rare and frequently suppressed due to confidentiality rules. Death records were included if AF was listed as the underlying cause of death (International Classification of Diseases, 10^th^ Revision (ICD-10): I48) and COPD was recorded as a contributing cause using ICD-10 codes J40-J44, which encompass chronic bronchitis and emphysema subcategories [11]. To maintain analytical clarity and comparability with prior AF-specific mortality studies, only deaths with AF as the underlying cause were considered, while those with reversed coding order (COPD as underlying and AF as contributing) were excluded. Each record represented a unique death certificate, and duplicates were automatically excluded by the CDC WONDER system.

Demographic variables included sex (male, female) and race/ethnicity based on the CDC’s bridged-race classification, which categorizes decedents as White, Black or African American, American Indian or Alaska Native, or Asian or Pacific Islander. Hispanic ethnicity, although available in the dataset, was not analyzed separately due to incomplete reporting and frequent suppression of small cell counts (<10), which may lead to unreliable estimates. Geographic variables were categorized according to the 2013 National Center for Health Statistics (NCHS) Urban-Rural Classification Scheme for Counties [12], which divides counties into large central metro, large fringe metro, medium metro, small metro, micropolitan, and non-core rural areas. The place of death was categorized as a medical facility, home, hospice, or nursing facility as reported in the MCD dataset. The 2013 NCHS urban-rural classification [12] was applied retrospectively to ensure consistency in categorization across the study period despite evolving census definitions.

Mortality rates were reported as both crude and age-adjusted mortality rates (AAMRs) per 1,000,000 population. All rates were standardized to the 2000 U.S. Standard Population, following CDC/NCHS methodology to enable valid temporal comparisons. Suppression of cells with fewer than 10 deaths was automatically enforced in accordance with the CDC confidentiality policy. No imputation or aggregation was performed for suppressed values to preserve data integrity, although this may have limited trend assessment for smaller subgroups.

Temporal mortality trends were analyzed using the Joinpoint Regression Program (version 5.4.0, April 2025) developed by the National Cancer Institute, Bethesda, MD. Log-linear regression models were fitted to the natural logarithm of annual AAMRs to estimate the annual percent change (APC) and corresponding 95% confidence intervals (CIs). A maximum of two joinpoints (three trend segments) was permitted, with the optimal model selected using the Monte Carlo permutation test and a global significance level of α = 0.05. Separate models were constructed for each sex, racial, and geographic subgroup using identical parameters. The Joinpoint software was chosen to ensure consistency with current CDC and NCI statistical methodologies while maintaining comparability with prior mortality trend analyses.

All data analyses adhered to Strengthening the Reporting of Observational Studies in Epidemiology (STROBE) guidelines for cross-sectional studies, ensuring transparency, reproducibility, and completeness. The dataset used is publicly accessible, and all parameters (ICD-10 codes, age criteria, and modeling methods) allow full replication of results.

## Results

From 1999 to 2020, the CDC MCD database recorded 37,738 deaths in the United States among individuals aged 25 years and older. Among these, deaths where AF (ICD-10: I-48) [11] was listed as the underlying cause of death and COPD (ICD-10: J44) [11] was recorded as multiple causes of death were included in the study (37,738). The crude mortality rate for AF with COPD as a contributing cause was 8.4 per 1,000,000 population. Deaths due to causes other than these criteria were excluded.

Demographic characteristics

Among the total deaths analyzed, males accounted for 17,267 (45.8%), while females accounted for 20,471 (54.2%). The mortality rate for AF with COPD as a contributing cause was higher in females compared to males, indicating a potential demographic disparity. Regarding racial distribution, the highest proportion of deaths occurred among White individuals (n = 35,666, 94.5%), followed by Black or African American individuals (n = 1,602, 4.2%), Asian or Pacific Islander individuals (n = 324, 0.9%), and American Indian or Alaska Native individuals (n = 145, 0.4%). The mortality burden was highest among White individuals, highlighting racial disparities in mortality trends related to AF and COPD.

Geographic characteristics

A majority of deaths occurred in metropolitan areas (n = 29,534, 78.26%), while non-metropolitan areas accounted for 8,204 (21.74%) of deaths. Regarding the place of death, most deaths occurred in medical facilities (n = 15,590, 41.31%), followed by nursing homes or long-term care facilities (n = 9,686, 25.67%), decedents’ homes (n = 9,497, 25.17%), and hospice facilities (n = 1,575, 4.17%).

Overall temporal trends

**Figure 1:**
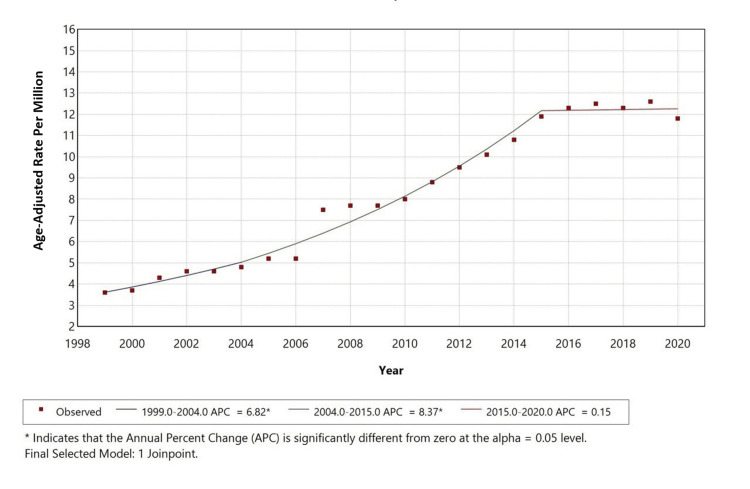
Overall age-adjusted mortality rates among adults aged 25+ in the United States from 1999 to 2020.

From 1999 to 2020, the AAMR for AF with COPD as a contributing cause initially increased with an APC of 6.23% (p<0.05) from 1999 to 2003. This was followed by a steeper increase from 2003 to 2015, with a statistically significant APC of 8.14% (p<0.05). From 2015 to 2020, the trend leveled off with a non-significant slight decrease with an APC of -0.13% (p<0.05), as shown in Figure 1.

Gender-specific trends

**Figure 2:**
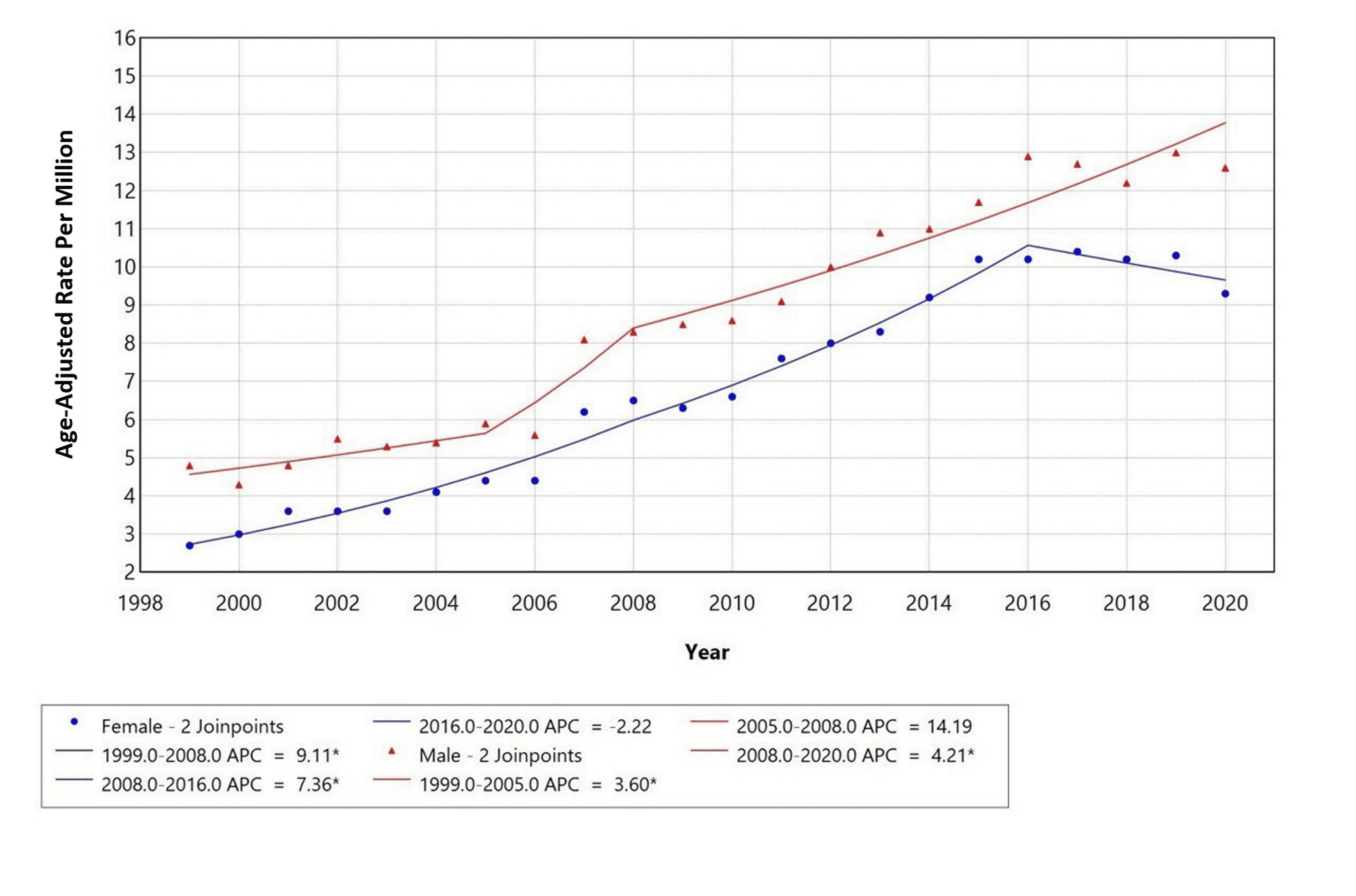
Trends in sex-stratified age-adjusted mortality rates among adults aged 25+ in the United States from 1999 to 2020. APC: annual percentage change

When stratified by gender, males had a higher AAMR (range ~4.5 to 14) compared to females (range ~2.5 to 10.5). However, both groups exhibited different trends with different APCs across time periods. Among females, the APC was 9.11% from 1999 to 2008 and 7.36% from 2008 to 2016, followed by a decline of -2.22% from 2016 to 2020. In contrast, males showed an APC of 3.60% from 1999 to 2005, followed by a sharp increase with an APC of 14.19% from 2005 to 2008, and then a continued increase with an APC of 4.21% from 2008 to 2020. Females showed a significant increase followed by a slight decline in mortality during 2016 to 2020, while a consistent upward trend was observed in males throughout the period, as represented in Figure 2.

Race-specific trends

**Figure 3:**
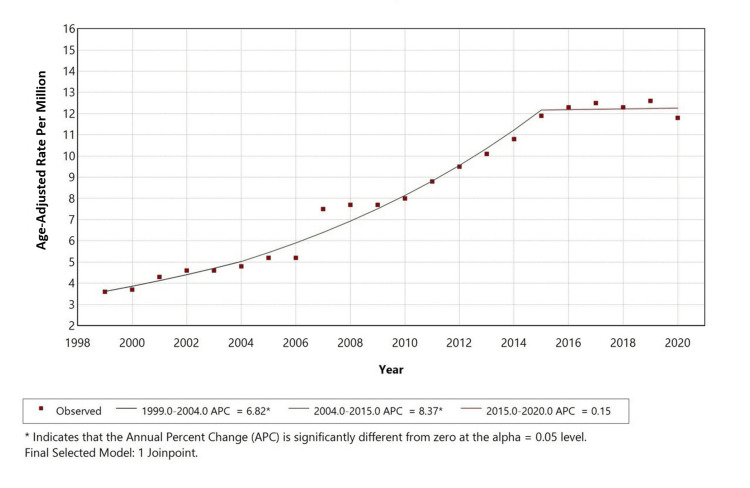
Trends in age-adjusted mortality rates among White adults aged 25 years and older in the United States from 1999 to 2020.

From 1999 to 2020, AAMR for White individuals in the United States initially increased sharply with an APC of 6.82% between 1999 and 2004. This upward trend continued and steepened from 2004 to 2015 with a statistically significant APC of 8.375%. From 2015 to 2020, the rate stabilized with a non-significant slight increase with an APC of 0.15%. These changes were statistically significant except for the final period. Data for other racial groups were not reported due to suppression for counts < 10, preventing reliable analysis of trends in those populations, as shown in Figure 3.

## Discussion

This retrospective analysis of U.S. mortality (1999-2020) among adults ≥25 years, using the CDC MCD database, examined deaths with AF (ICD-10 I48) as the underlying cause and COPD (ICD-10 J44) as a contributing cause. We identified 37,738 such deaths, with a crude mortality rate of 8.4 per 1,000,000 population and AAMRs that increased significantly from 1999 to 2015 (APC 6.23% and 8.14% in successive segments), followed by stabilization from 2015 to 2020 (APC −0.13%, non-significant). Females accounted for a higher proportion of total deaths, whereas males had higher AAMRs, and White individuals contributed the largest absolute number of deaths. Most fatalities occurred in metropolitan areas, yet several rural strata showed higher proportional (age-adjusted) mortality, underscoring geographic disparities.

Mechanistically, the observed epidemiology aligns with established pathophysiology. COPD-related hypoxia and systemic inflammation (e.g., HIF-1α/VEGF activation with downstream MMP-9/TGF-β1 signaling) and hypercapnia-related electrophysiologic alterations support atrial remodeling and arrhythmogenesis, while pulmonary hypertension and right-sided chamber enlargement increase atrial stretch and ectopy [6, 10, 11, 14, 15]. These pathways-derived primarily from experimental models and small human studies-provide biological plausibility for the higher AF-related mortality when COPD coexists, without implying causality at the population level.

Sex differences likely reflect a combination of demography, care patterns, and biology. Although males exhibit higher AAMRs, females account for more deaths overall, consistent with greater longevity and a larger elderly female population at risk. Prior work indicates that women with AF may face higher risks of adverse outcomes and potential under-recognition or undertreatment, which could contribute to the higher absolute burden among females despite lower standardized rates [1,7,16]. Racial patterns also warrant careful interpretation. White individuals contribute most deaths in absolute terms, given population composition; however, rate-based comparisons are more informative. Evidence suggests AF may be under-detected in Black, Hispanic, Asian, and American Indian populations despite a higher burden of risk factors (e.g., hypertension, obesity, diabetes), potentially due to differential access to monitoring and specialty care; higher case fatality in some subgroups of Black populations has been reported despite lower recorded AF prevalence [1,3,17].

Geographically, metropolitan counties account for most deaths, yet non-metropolitan areas show disproportionately higher AAMRs in several strata, consistent with greater exposure to tobacco and environmental/occupational hazards, fewer specialists, and challenges in infrastructure (e.g., hospital closures, workforce shortages) [4,5]. Emphasizing AAMRs rather than raw counts avoids confounding by population size and clarifies that rurality is associated with higher proportional mortality even when absolute deaths are fewer.

The post-2015 plateau most plausibly reflects stabilization rather than decline. Potential contributors include diffusion of evidence-based AF stroke prevention and COPD management, maturation of coding practices, and demographic dynamics reaching a steady state. Notably, national data indicate AF/atrial flutter mortality in older adults roughly doubled over 1999-2020 despite modern therapies [18], while overall COPD+cardiovascular mortality changed little [19], suggesting that the AF+COPD subgroup disproportionately drives the observed increase. Persisting gaps, such as incomplete risk-factor control, underuse of anticoagulation or pulmonary therapies, and structural barriers to care, may blunt therapeutic gains and help explain the residual burden.

These findings have clear public-health implications. Reducing mortality in AF with COPD will require integrated cardiopulmonary care, systematic attention to equity (sex, race/ethnicity, rurality), and policy-level interventions that expand rural infrastructure, ensure equitable access to screening and specialty services, and support smoking cessation and pulmonary rehabilitation. Future work linking vital statistics with clinical and administrative data could disentangle biological from structural drivers and better characterize under-detection, treatment patterns, and outcome differences across subgroups.

Limitations

This study has several important limitations. First, it relies on death certificate data, which are subject to misclassification of underlying vs. contributing causes and secular changes in reporting. Second, the analysis is limited to deaths with AF as the underlying cause and COPD as contributing; reversing the coding order would define a distinct cohort and may lead to conservative estimates of the overall joint burden. Third, suppression rules (<10 deaths) constrain subgroup trend estimation for smaller populations and may attenuate apparent disparities. Fourth, the MCD database lacks individual-level detail on smoking, treatment, disease severity, and socioeconomic status; thus, residual confounding is likely. Fifth, molecular mechanisms cited (e.g., HIF-1α, MMP-9) are based largely on experimental/biomarker evidence rather than large human cohorts and are presented for contextual plausibility rather than causal inference. Finally, results reflect U.S. mortality and may not generalize to other healthcare systems with different access, coding, and environmental profiles. Despite these constraints, national coverage, age-standardized rates, and consistent analytic methods provide a robust depiction of long-term trends and disparities in AF-COPD mortality.

## Conclusions

Mortality from AF with COPD as a contributing cause increased significantly from 1999 to 2015 before stabilizing, with disparities by sex, race, and geography. Women, White individuals, and metropolitan residents carried the highest burden. These findings highlight the need for early recognition, integrated cardiopulmonary care, and targeted prevention strategies. Strengthening healthcare access, particularly in underserved areas, and addressing systemic inequities are essential to reduce mortality and improve outcomes in this high-risk population.
